# Improvement Needed: Cancer Pain Management in 2012

**Published:** 2012-07-01

**Authors:** Pamela Hallquist Viale

In 32 years of practice, I have seen vast improvement in the treatment of patients with cancer. From the introduction of novel therapeutic treatments to the approval of innovative pain medications, our patients with cancer have benefited from the approval of new therapies. I eagerly anticipated new forms of medications for pain, as sustained-release forms of old standards offered better pain relief and easier administration for our patient population. The introduction of the fentanyl patch and sustained-release oxycodone was welcomed in my practice, as we sought to obtain better pain relief for our patients. National and federal guidelines reported new pain management standards over two decades ago, bringing heightened awareness of this debilitating symptom to the health-care professional (HCP). And yet, despite the potential for improved pain management made possible by the new drug formulations and inventive new agents, patients still report pain during their experience with cancer.

## Prevalence of Undertreatment

There are myriad reasons for possible undertreatment of pain in our population of patients with cancer. A pivotal paper published in 1994 reported that pain is the most persistent and devastating symptom for patients with advanced disease and that both HCPs and patients described this symptom as often poorly managed (Cleeland et al., 1994). The authors of this paper conducted a study of 1,308 patients and determined that although multiple factors play a role in predicting inadequate pain management, 42% of their study participants were not given adequate analgesic therapy. Centers treating minorities had even higher rates of poor pain control, as patients were three times more likely to report inadequate management (Cleeland et al., 1994).

The prevalence of pain in patients with cancer remains significant. A meta-analysis published in 2007 reported that cancer pain remains a major concern for this population of patients (van den Beuken-van Everdingen et al., 2007). Patients with advanced disease had the highest incidence (64%), but 59% of patients on anticancer therapies and 33% of patients who received curative therapy also reported pain. Over one-third of the patients in the study graded their pain as moderate or severe (van den Beuken-van Everdingen et al., 2007). The authors concluded that despite the international recommendations from the World Health Organization, pain remained a serious concern for these patients. Deandrea and colleagues confirmed that patients with cancer report undertreatment of pain; in a review of 44 studies, the authors noted that nearly one of two patients with cancer is undertreated for pain (Deandrea, Montanari, Moja, & Apolone, 2008).

## A Persistent Problem

The most recent study to examine prescribing of pain medications to medical oncology patients with breast, colorectal, lung, or prostate cancer was published in the *Journal of Clinical Oncology* (Fisch et al., 2012). The prospective, observational study involved 3,123 ambulatory patients who completed a 25-item measure of pain, functional interference, and additional symptoms at the time of initial assessment and 4 to 5 weeks later. In this study, 67% of the patients reported having pain or needing analgesics at the initial assessment; of these patients, 33% were receiving inadequate analgesic prescribing. There was no difference in the adequacy of analgesic therapy noted between the initial and follow-up visits (Fisch et al., 2012).

## Future Directions

Increasing awareness and education regarding this potentially devastating symptom continues to be one of the approaches toward improvement of pain management in the cancer patient population; national and international guidelines and recommendations form the foundation for policies and procedures regarding optimal pain management for patients with cancer. Pain relief depends on appropriate management by health-care providers; these individuals must provide adequate care and pain management using the best principles and evidence-based practice information available to them (Paice & Ferrell, 2011).

Advanced practitioners (APs) working in pain and symptom management are helping to forge the way; however, all APs must continue to reinforce these principles and examine their own practices for management of cancer pain. Understanding of the common barriers to adequate pain management is important; comprehensive assessment and understanding of pain syndromes and possible therapies are essential (Paice & Ferrell, 2011). Better communication between patients and their health-care providers remains a critical part of the plan for optimal pain management (Fisch et al., 2012).
